# Quantum mechanics insights into melatonin and analogs binding to melatonin MT_1_ and MT_2_ receptors

**DOI:** 10.1038/s41598-024-59786-x

**Published:** 2024-05-13

**Authors:** Gabriela de Lima Menezes, Katyanna Sales Bezerra, Jonas Ivan Nobre Oliveira, John Fontenele Araújo, Douglas Soares Galvão, Roosevelt Alves da Silva, Marielena Vogel Saivish, Umberto Laino Fulco

**Affiliations:** 1 Departamento de Biofísica e Farmacologia, Universidade Federal do Rio Grande no Norte, Natal, RN 59072-970 Brazil; 2 Bioinformatics Multidisciplinary Environment, Programa de Pós Graduação em Bioinformática, Universidade Federal do Rio Grande do Norte, Natal, RN 59078-400 Brazil; 3 Departamento de Fisiologia e Comportamento, Universidade Federal do Rio Grande no Norte, Natal, RN 59072-970 Brazil; 4 Applied Physics Department, University of Campinas, Campinas, São Paulo, 13083-859 Brazil; 5 Unidade Especial de Ciências Exatas, Universidade Federal de Jataí, Jataí, GO 75801-615 Brazil; 6 Laboratório de Pesquisas em Virologia, Departamento de Doenças Dermatológicas, Infecciosas e Parasitárias, Faculdade de Medicina de São José Do Rio Preto, São José Do Rio, Preto, SP 15090-000 Brazil; 7 Centro Nacional de Pesquisa em Energia e Materiais (CNPEM), Brazilian Biosciences National Laboratory, Campinas, SP 13083-100 Brazil

**Keywords:** Proteins, Sleep disorders, Cheminformatics

## Abstract

Melatonin receptors MT_1_ and MT_2_ are G protein-coupled receptors that mediate the effects of melatonin, a hormone involved in circadian rhythms and other physiological functions. Understanding the molecular interactions between these receptors and their ligands is crucial for developing novel therapeutic agents. In this study, we used molecular docking, molecular dynamics simulations, and quantum mechanics calculation to investigate the binding modes and affinities of three ligands: melatonin (MLT), ramelteon (RMT), and 2-phenylmelatonin (2-PMT) with both receptors. Based on the results, we identified key amino acids that contributed to the receptor-ligand interactions, such as Gln181/194, Phe179/192, and Asn162/175, which are conserved in both receptors. Additionally, we described new meaningful interactions with Gly108/Gly121, Val111/Val124, and Val191/Val204. Our results provide insights into receptor-ligand recognition’s structural and energetic determinants and suggest potential strategies for designing more optimized molecules. This study enhances our understanding of receptor-ligand interactions and offers implications for future drug development.

## Introduction

Melatonin (N-acetyl-5-methoxytryptamine) is a neurohormone secreted during sleep that was first isolated from melanocytes of frogs and fish by Lerner et al.^1,2^. In 1993, it was discovered that melatonin is secreted by the pineal gland of all vertebrates and is involved in regulating circadian rhythms^3^. The secretion of melatonin is controlled by the suprachiasmatic nucleus (SCN) in the hypothalamus^4^.

The SCN exerts control over the efferents of the paraventricular nucleus of the hypothalamus, modulating most of the circadian functions of the autonomic nervous system, including the sympathetic pathway whose preganglionic neurons are located in the intermediate lateral column of the spinal cord and which projects to the postganglionic neurons of the superior cervical ganglion. In this pathway, the SCN rhythmically controls the release of noradrenaline, which promotes the activation of the enzyme arylalkylamine N-acetyltransferase (AA-NAT) and consequently the production of melatonin by the pineal gland^5^. In the case of melatonin, its secretion is suppressed by light and stimulated in a dark environment^6^. We also know that melatonin has been reported to be secreted by other organs such as the retina^7–9^, gastrointestinal tract^10^, skin^11^, lymphocytes^12^, and bone marrow^13^. However, the function of melatonin produced outside the pineal does not have a great functional role. It is only a result of the enzymatic degradation of serotonin.

Two subtypes of melatonin receptors have been described: MT_1_^14^ and MT_2_^15^, both of which are G protein-coupled receptors (GPCRs). A third receptor, MT_3_, was later characterized as a reductase enzyme involved in oxidative stress protection events. The localization of MT_1_ and MT_2_ is not homogeneous. MT_1_ is found in many organs and tissues, such as the SCN^16,17^, cerebellum^18^, ovary^19^, testis^19^, liver, kidney, and others^20^. On the other hand, MT_2_ is more restricted to the brain, although it has been observed in other tissues such as the lung, heart, and aorta^16^. MT_1_ and MT_2_ have 350 and 362 amino acids, respectively. They also have 55% sequence homology, and this value increases to 70% when only the transmembrane (TM) region is considered. In terms of function, MT_1_—which has a higher affinity for melatonin—has been shown to acutely suppress neuronal firing, whereas MT_2_ is important for efficient phase-shifting^21^.

Structurally, they are also very similar (C*α* RMSD < 0.6 Å) and the residues interacting with the binder are conserved^22,23^. Both receptors have the described GPCR configuration: seven transmembrane helices (TM1 to TM7) with an extracellular N-terminus, three extracellular loops (ECL), three intracellular loops (ICL), and a short amphipathic helix VIII, oriented parallel to the membrane^25^. It has been suggested that access to the orthosteric binding site (recognized by melatonin) is via the IV and V helices, which open toward the lipid bilayer^25^.

Currently, there is a high prevalence of sleep disorders in the general population, often associated with work shifts, travel, and mental illnesses such as depression and anxiety^24^. One of these sleep disorders is insomnia, defined as difficulty in initiating or maintaining sleep or constant episodes of non-restorative sleep. It has been described that insomnia affects approximately 30–35% of the adult population and has been associated with an increased risk of mortality. This condition affects both physically and economically those who have it, whether by frequent expenses with doctors and medications or reduction in the working and studying hours due to fatigue^27^. Moreover, studies have linked insomnia with the reduction of cognitive capacity, affecting the retention and manipulation of working memory, problem-solving, and episodic memory^25^. Beyond the cognitive issues, evidence suggests that insomnia increases the risks of developing high blood pressure and type 2 diabetes mellitus^26–28^. Therapeutic interventions for insomnia include both pharmacologic and nonpharmacologic ones, with the latter including lifestyle and behavioral changes such as physical activity, sleep hygiene, sleep restriction, and relaxation therapies^29,30^.

Pharmacological treatment can be divided into two groups of compounds: Benzodiazepines (BZD) and non-benzodiazepines (non-BZD), both of which are sedative-hypnotics.

The BZDs (Estazolam, Flurazepam, Quazepam, or Triazolam) are agents that increase total sleep time and quality of sleep. However, because of adverse effects, such as next-day hangover, amnesia, and potential overuse, these drugs are controversial for long-term treatment of insomnia^31,32^. The non-BZDs (including Zolpidem, Zaleplon, and Eszopiclone) are not as effective as BZDs in improving sleep quality or efficiency, but they are more effective in inducing sleep^33^. In addition, Zolpidem use is associated with other adverse effects such as daytime sleepiness, hallucination, dizziness, headache, nausea, vomiting, and the potential for abuse and dependence^34^.

Despite the adverse effects of BZD and non-BZD, there is increasing interest in the therapeutic use of melatonin to treat insomnia, especially as a sleep inducer. However, the use of melatonin as a medicine has yet to be regulated in several countries, but it is widely used as a food supplement. For example, exogenous melatonin is one of the most popular natural products taken by adults in the United States^35^. The American Academy of Sleep Medicine recommends melatonin for the treatment of circadian rhythm sleep disorder: advanced sleep phase type, free-running (none trained) type in blind adults, and irregular sleep–wake type in children and adolescents with neurological disorders^36^. However, because of its rapid absorption and very short half-life elimination (t_1/2_) from only 40–60 min^37^, it has been suggested that melatonin works best as a chronobiotic rather than a hypnotic^38^, that is, melatonin is effective in regulating the sleep–wake cycle.

To enhance the effectiveness of supplemental melatonin, the production of prolonged-release melatonin—which has a longer duration of action than regular melatonin—emerged as an option. Research has shown that prolonged-release melatonin can increase the sleep duration of people with insomnia^39^. Another way is to use melatonin agonists and analogs, which are compounds that mimic or modify the effects of melatonin on its receptors. Melatonin agonists and analogs have different structures. However, most share a common pharmacophore: an amide group attached to an aromatic ring with a methoxy or a similar group, such as bromine. Some analogs have additional substitutions at the C2 position of the indole ring, which can enhance their affinity for the melatonin receptors by up to 10 times compared to melatonin itself. Examples of these analogs include 2-iodomelatonin and 2-bromomelatonin, which have halogen atoms, 2-phenylmelatonin (2-PMT), which has an aromatic group, and ramelteon (RMT)^40–42^.

Ramelteon^43^ was the first melatonin receptor agonist approved by the U.S. Food and Drug Administration (FDA) to treat insomnia associated with difficulty in falling asleep. Compared to melatonin, RMT has six times more affinity for MT_1_ receptors and about four times more affinity for MT_2_ ones^44^, suggesting that this drug is more suitable for the treatment of insomnia associated with problems in falling asleep^43^. Indeed, studies have shown that taking RMT shortens sleep latency and increases total sleep time and efficiency compared to placebo^45^.

The metabolization of RMT produces four metabolites: M-I, M-II, M-III, and M-IV. In serum, M-II is more abundant than RMT^46,47^. Although M-II exhibits a lower affinity for MT_1_ and MT_2_ receptors compared to RMT, it also has a longer half-life than melatonin^47^. Overall, the extended half-life of both RMT and its major metabolite, M-II, contributes to their enhanced efficacy compared to melatonin^44^.

RMT also overcomes the adverse effects observed with BZD and non-BZD medications. In the phase I studies, only 5% of the patients discontinued RMT compared to 2% in the placebo group. In phase III, the associated adverse effects were somnolence (0.8%), dizziness (0.5%), nausea (0.3%), fatigue (0.3%), headache (0.3%), and insomnia (0.3%), as described in the manufacturer’s prescribing information^48^. In addition, no abuse or dependence potential and no effects on behavior or cognitive performance were observed for RMT^49–52^. Although RMT is an excellent melatonin agonist, it has been used in silico studies to obtain new drugs through binder-based drug design. It has also been considered for treating diseases in which melatonin receptor ligands are currently under investigation, including cancer, obesity, diabetes, and pain^53^.

In this context, in silico studies have been described as an important initial step in the development of new, more potent drugs based on existing ones^54,55^. These studies involve the analysis of protein–ligand interactions using energetic calculations methodologies through classical mechanics (MM), quantum mechanics (QM), or hybrid methods (QM/MM)^56–59^. This allows for the evaluation of the strengths and weaknesses of each ligand, proposing structural modifications that enhance its affinity for the receptor.

Molecular dynamics (MD) simulations using calculations based on classical mechanics are widely used to obtain optimized structural models of receptor-ligand complexes that are not available in the Protein Data Bank (PDB), especially those obtained through in silico molecular docking^60,61^. From the ensemble of conformations generated in MD, protein–ligand interaction calculations can be obtained with relatively low computational cost using methodologies such as Quantum- Mechanical/Molecular-Mechanical Generalized Born Surface Area (QM/MM-GBSA), which is based on the use of semi- empirical functional for quantum mechanics calculations, and classical field force for molecular mechanics calculations.

In this case, semi-empirical methods are reduced-order Hamiltonians in which some elements are replaced by empirical parameters adjusted to experimental or ab initio data. Examples include the PM3, PM6, and AM1 models, among others^62,63^. Always aiming for increased accuracy and reduced computational cost, semi-empirical functionals are applied to only a part of the complex (usually defined by the ligand distance), while the remaining is calculated using classical force fields. Despite being a methodology used in drug development studies^64^, it is known that its accuracy is still lower, especially when compared to purely ab initio methods such as Density Functional Theory (DFT)^65^.

The DFT emerged as a quantum functional to address the limitations of semi-empirical approaches and other quantum methodologies, such as Hartree–Fock (HF), enabling the study of biological molecules^66,67^. Despite the advantages of DFT, it remains computationally demanding, requiring the use of high-performance clusters and other cost-reduction techniques. One such approach is molecular fragmentation with conjugated caps (MFCC), which involves splitting the system into smaller molecules^71^.

In this aspect, the present work investigates the interaction of two melatonin receptors (MT_1_ and MT_2_) complexes with RMT, 2-PMT, and MLT. For this purpose, the crystallographic data of the structural complexes and complexes obtained from docking simulations were used as input geometrical data in the calculation. The quantum binding energy calculations were performed using the DFT formalism DFT in association with the MFCC method. This approach allows us to determine the main contributions of amino acids to their affinity with melatonin receptors.

## Results

### Molecular docking and molecular dynamics simulation

**Figure 1:**
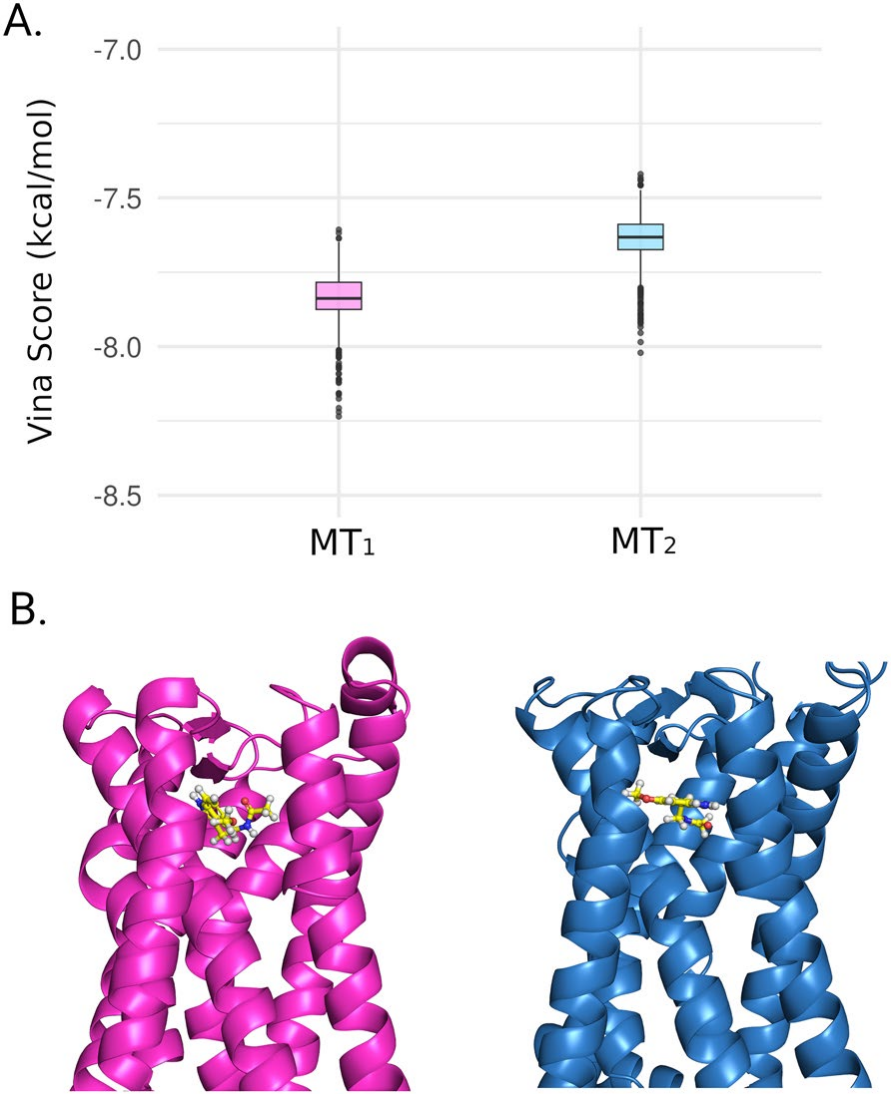
Melatonin docking in MT_1_ and MT_2_. (**A**) Box plot of Vina score distribution in kcal/mol. The pink box is related to MT_1_-MLT complex and the blue is related to MT_2_-MLT docking. (**B**) Best docking pose of MLT (yellow stick) bound in MT_1_ (pink cartoon) and MT_2_ (blue cartoon).

To obtain the MT_1_-MLT and MT_2_-MLT complexes, molecular docking was performed using AutoDock Vina^68^. The distribution of Vina scores (in kcal/mol) from 1000 rounds of docking can be observed in Fig. 1A, and a summary of the obtained values is presented in Table 1. The Vina scores for the MT_1_-MLT complex ranged from − 7.607 kcal/mol to − 8.235 kcal/mol, with a mean (SD) value of − 7.832 kcal/mol (0.08753). Similarly, for the MT_2_-MLT complex, the Vina scores ranged from − 7.420 kcal/mol to − 8.021 kcal/mol, with a mean (SD) of − 7.632 kcal/mol (0.07979). These results indicate that melatonin has a higher affinity for the MT_1_ protein compared to MT_2_. However, it is important to note that the primary objective of the exhaustive Vina docking is to rank the complexes based on Vina scores. Furthermore, as depicted in Fig. 1B, the best MLT conformations bind to the same pocket as observed in experimental structures^23^, further confirming the reliability of the docking results. Therefore, the obtained data strongly suggest that the docking results are suitable for proceeding to MD simulations.

**Table 1:** Summary of Vina docking results based on 1000 ligand conformations.

Protein	Vina Score (kcal/mol)						
	Min	1st quartile	Median	Mean	3rd. quartile	Máx	sd^†^
MT_1_	− 8.235	− 7.875	− 7.838	− 7.832	− 7.784	− 7.607	0.08753
MT_2_	− 8.021	− 7.674	− 7.632	− 7.640	− 7.589	− 7.420	0.07979

^†^sd: standard deviation.

**Figure 2:**
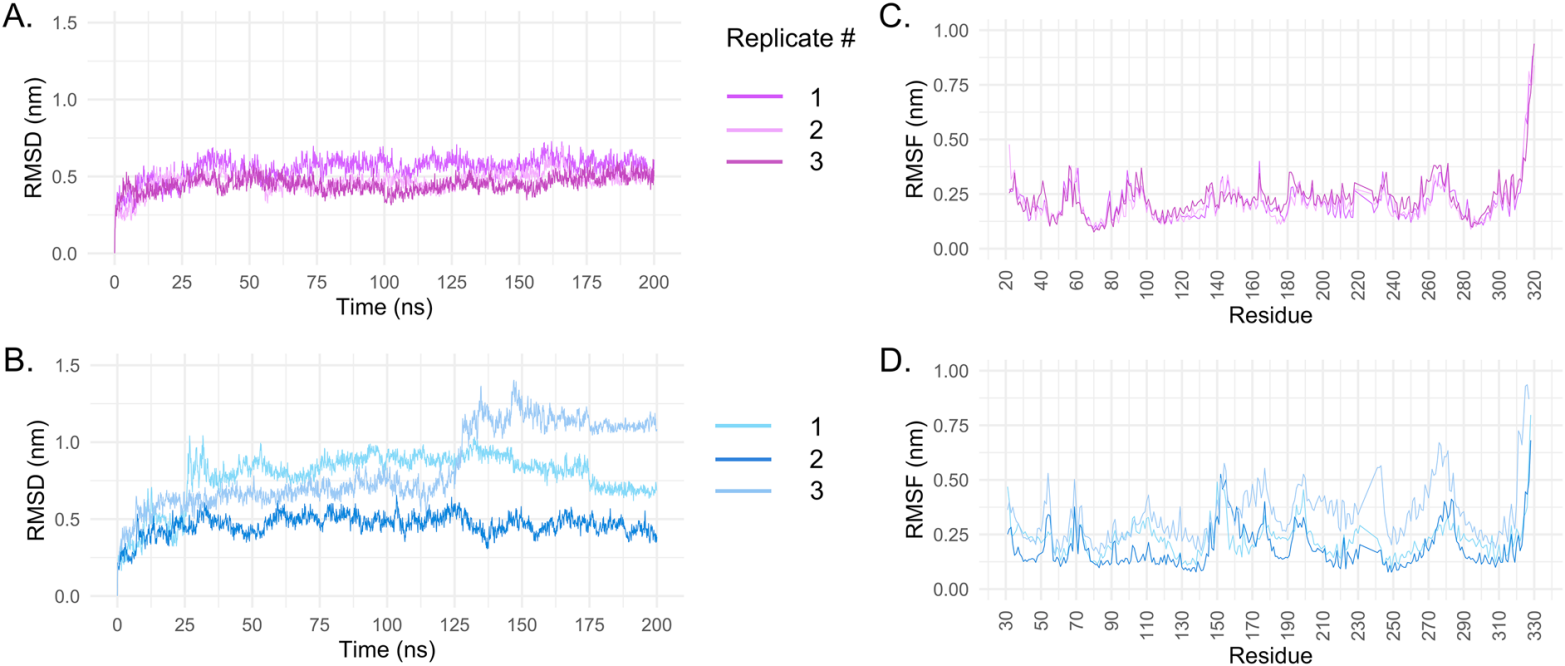
RMSD and RMSF analysis of 200 ns three MD trajectory. (**A**) and (**B**) RMSD plots in three replicates of MT_1_ and MT_2_ MD simulations, respectively. (**C**) and (**D**) RMSF plots in three replicates of MT_1_ and MT_2_ by residue number, respectively.

The main purpose of the MD simulations is to adjust the conformation of the protein and ligand in a solvent environment. The Root Mean Square Deviation (RMSD) is a metric that evaluates the difference between 3D structures based on atomic distances. A higher RMSD value indicates a greater difference in structures. In this study, we compared the structures along the trajectory with the initial frame. The RMSD of the MT_1_-MLT complex can be seen in Fig. 2A. It is noteworthy that all three replicates exhibited similar behavior throughout the 200 ns trajectory, with an RMSD value of approximately 0.5 nm. Additionally, the Root Mean Square Fluctuation (RMSF) analysis (Fig. 2C), which evaluates the average residue fluctuation, showed no significant differences among the three replicates. It is expected to observe higher fluctuation in the N- and C-termini of the protein. However, in this case, only higher residue fluctuation was observed in the C-terminal region of the protein.

Unlike the MT_1_-MLT complex, the three replicates of MT_2_-MLT exhibited distinct behaviors along the MD trajectory. Among them, the RMSD (Fig. 2B) of replicate 2 was more stable. Like MT_1_-MLT, the RMSD value remained around 0.5 nm. In contrast, replicate 3 showed a higher but stable RMSD (0.65 nm) until 125 ns into the simulation. Afterward, the RMSD increased to values higher than 1.1 nm, and after 150 ns, stability was observed near this RMSD value. The heatmap plot of RMSD per residue over time (Supplementary Figure S1C) revealed an increasing fluctuation of residues attached in the MT_2_ N-terminal, which corresponds to the apocytochrome BRIL (UniProt P0ABE7) from *Escherichia coli*, after 125 ns. Additionally, around 60 ns, an increased RMSD in residues of MT_2_ C-terminal corresponding to another fusion protein, rubredoxin (Rub, UniProt P00268). Notably, these residues are distant from the ligand binding pocket, suggesting that they have insignificant influence on the ligand binding mode. Finally, replicate 1 showed intermediate fluctuations with values near 0.8 nm until 175 ns, followed by a small decrease to values near 0.70 nm. The fusion protein also observed these variations, as shown in Supplementary Figure S1A. The structure of MT_2_ and the fusion proteins can be seen in Supplementary Figure S2. The RMSF analysis of the MT_2_ replicas (Fig. 2D) indicates that the fluctuation patterns were quite similar among them, despite the amplitude of replica 3 being larger, which corroborates with the observations made in the RMSD graph. Conversely, the fluctuations of replicas 1 and 2 were alike in terms of both amplitude and fluctuation pattern.

The RMSD analysis of the ligand is an interesting means to evaluate if the ligand remained in the binding pocket or if displacement occurred. A high RMSD indicates a modification in the ligand’s position. Supplementary Figure S3 shows that MLT in complex with MT_1_ and MT_2_ remained in the pocket as the RMSD did not show high values. However, MLT in MT_1_ showed an RMSD near 0.5 nm, while MLT in MT_2_ showed an RMSD near 0.8 nm, suggesting that MLT-MT_2_ underwent a higher conformational change during the MD simulation compared to MLT-MT_1_. These results were found to be suitable for performing the QM/MM-GBSA analysis.

**Figure 3:**
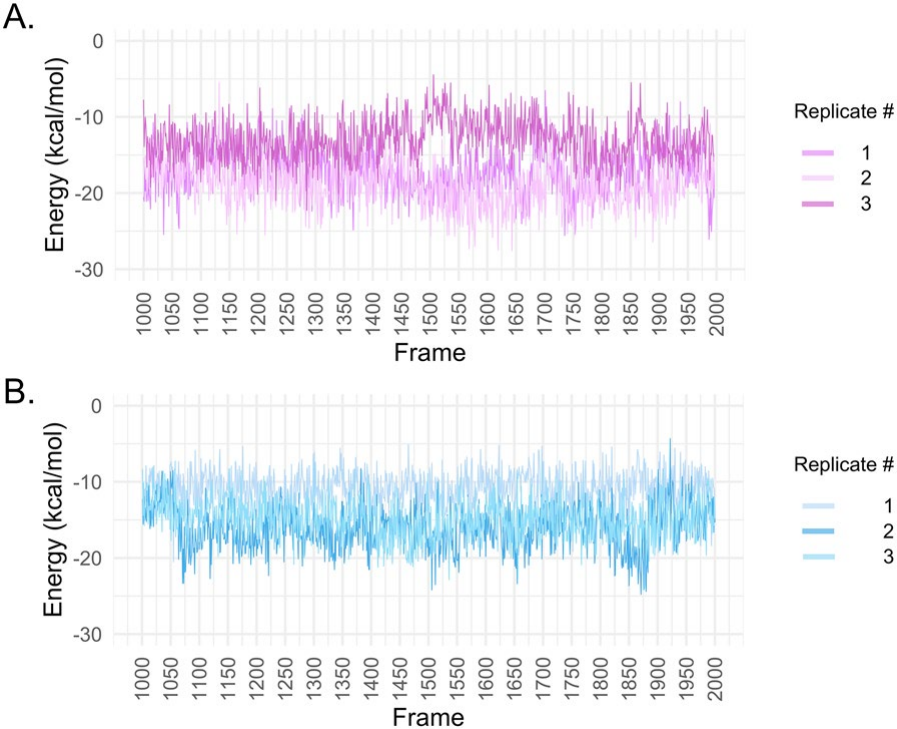
QM/MM values along trajectory frames (**A**). MT_1_ three replicates. (**B**) MT_2_ replicates.

The results of QM/MM-GBSA can be observed in Table 2 and Fig. 3. In Table 2, it is possible to observe that the mean of all replicas showed lower energy in the interaction with MLT for MT_1_ compared to MT_2_, except for replica 3. This is also true when we compare the lowest value for each replicate. Notably, the QM/MM-GBSA analysis of MT_1_-MLT (Fig. 3A) showed more variation among replicates compared to the MT_2_-MLT complex. However, both complexes exhibited values mostly lower than − 10 kcal/mol. As observed in the Vina docking results, the complex with higher affinity was MT_1_-MLT. The selected frames for MFCC/DFT analysis were frame 1648 (replicate 2, MT_1_), which presented − 27.57 kcal/mol of binding energy, and frame 1871 (replicate 2, MT_2_) which presented − 24.78 kcal/mol of energy. Now, there are six complexes for which QM calculations will be performed: MT_1_-MLT, MT_2_-MLT, MT_1_-RMT, MT_2_-RMT, MT_1_-2-PMT, and MT_2_-2-PMT. The last four complexes were obtained from the Protein Data Bank (accession codes: 6ME2, 6ME9, 6ME3 and 6ME6 respectively).

**Table 2:** QM/MM results of MT_1_-MLT and MT_2_-MLT complexes.

_Protein_	Replicate 1		Replicate 2		Replicate 3	
	Mean ± sd^†^	Lowest value	Mean ± sd^†^	Lowest value	Mean ± sd^†^	Lowest value
MT_1_	− 17.07 ± 3.01	− 26.07	− 18.88 ± 2.94	− 27.57	− 13.00 ± 2.72	− 22.26
MT_2_	− 10.81 ± 2.23	− 16.92	− 16.08 ± 2.87	− 24.78	− 14.82 ± 2.60	− 22.84

**Figure 4:**
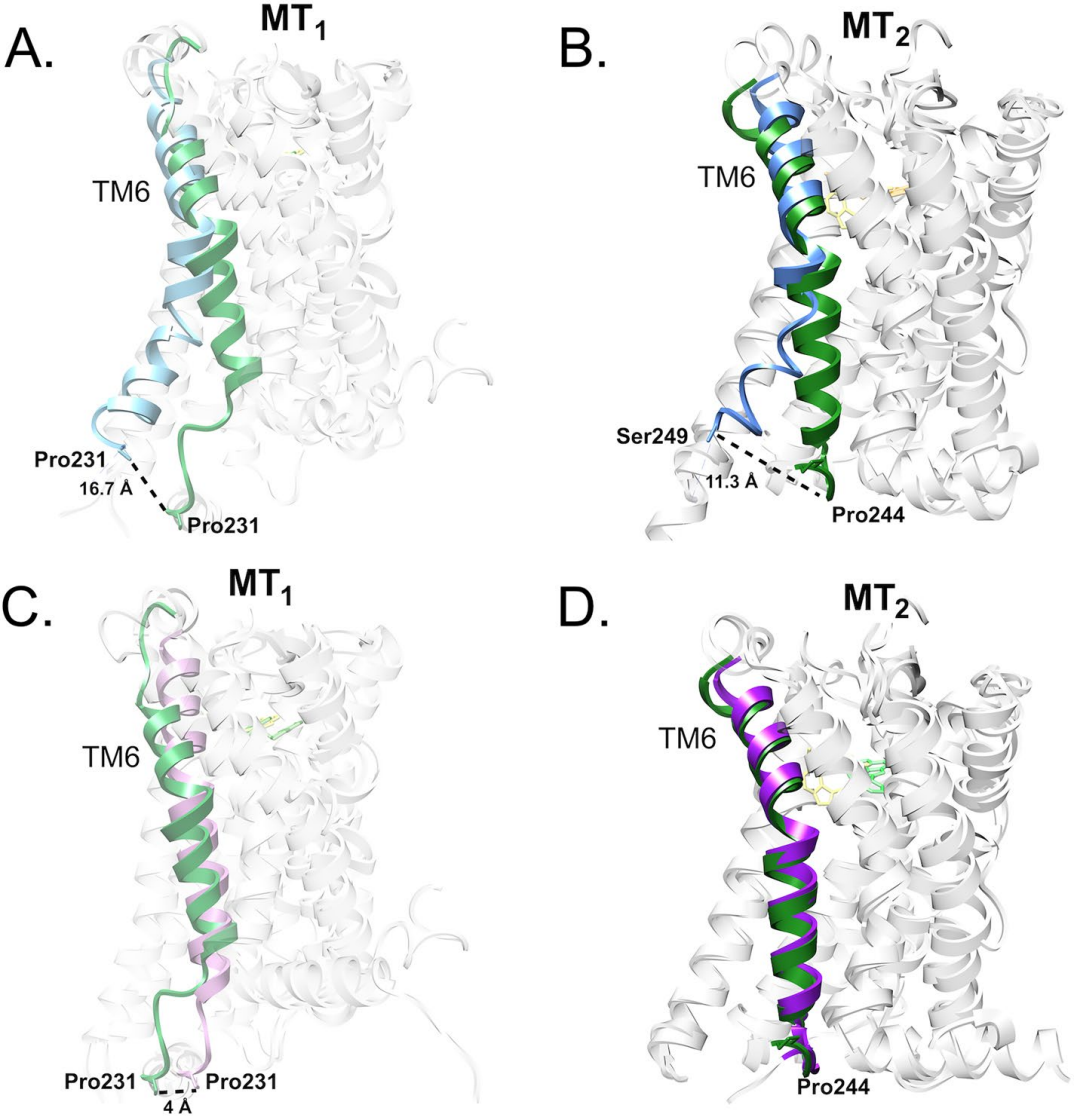
Comparison of the MT_1_ and MT_2_ structures in their active and inactive forms. (**A**) Comparison of the active MT_1_ structures (blue color ribbon) with the MT_1_-MLT complex (green color ribbon). (**B**) Comparison of the active MT_2_ (blue color ribbon) with the MT_2_-MLT complex (green color ribbon). (**C**) Comparison of the inactive MT_1_ structures (pink color ribbon) with the MT_1_-MLT complex (green color ribbon). (**D**) Comparison of the inactive MT_2_ structures (purple color ribbon) with the MT_2_-MLT complex (green color ribbon).

The structures of MT_1_-MLT and MT_2_-MLT selected for QM (DFT) calculations were compared with their experimentally resolved counterparts, both in their active and inactive forms. The structure of MT_1_-MLT was compared with structures PBD ID: 7DB6^73^ (active, Cryo-EM) and PDB ID: 6ME2^25^ (inactive, X-ray) and the complex MT_2_-MLT was overlaid with structures PDB ID: 7VH0^69^ (active, Cryo-EM) and 6ME6^23^ (inactive, X-ray). As observed in Fig. 4A, a displacement of TM6 in the active state (light blue ribbon) is noted when compared to MT_1_ with MLT (light green ribbon). The same is observed in MT_2_-MLT (Fig. 4B). Figure 4C and D present the overlay of MT_1_-MLT and MT_2_-MLT with inactive MT_1_ and MT_2_, respectively. It is noted that the displacement of TM6 when comparing the complexes with each other is much smaller, indicating that the complex with MLT is anchored in the inactive form of the melatonin receptor. This is expected since the docking was performed with the protein in this conformation. Therefore, it is concluded that the MD simulation maintained the complex in its inactive state.

### MFCC and quantum mechanical calculations

**Figure 5:**
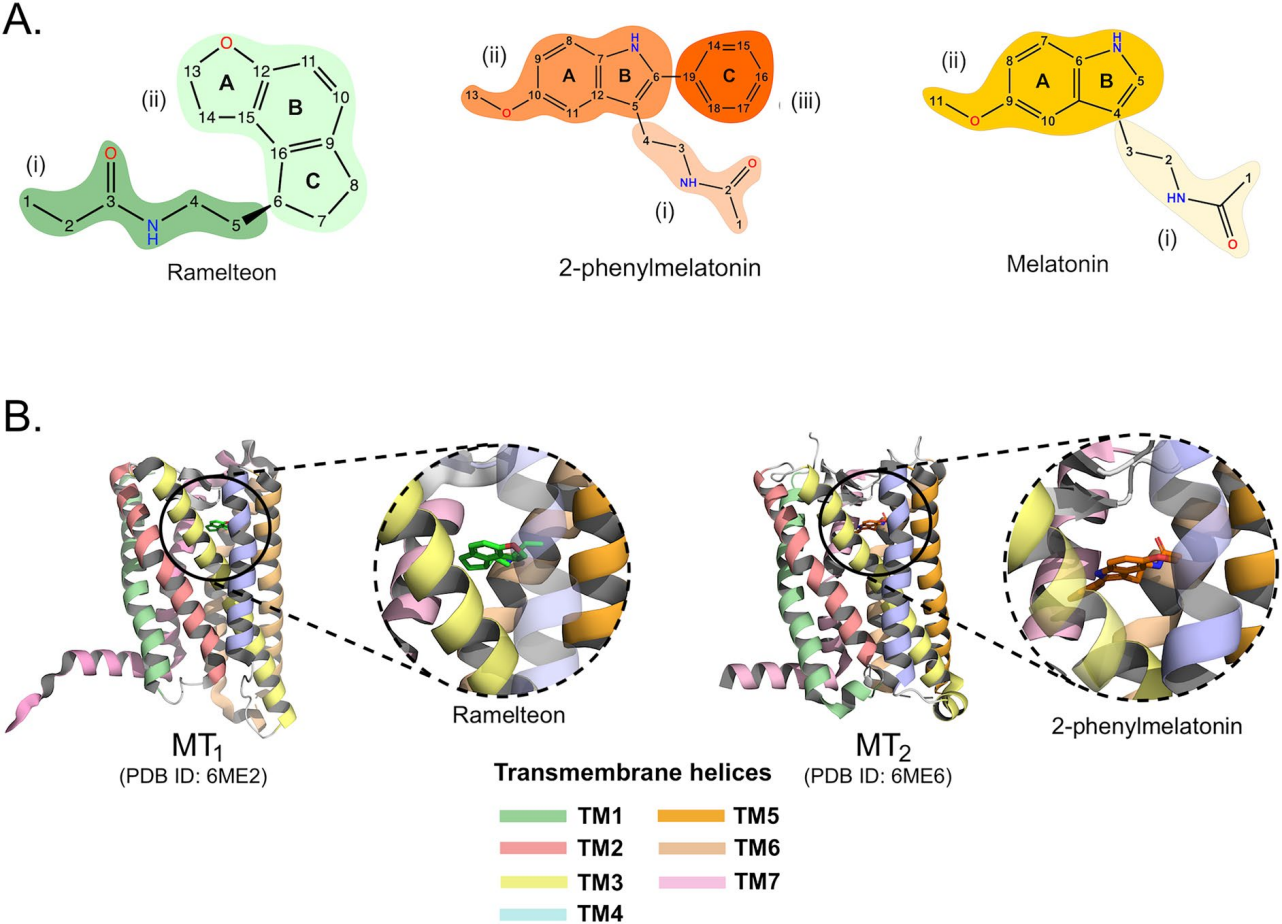
(**A**) Schematic representation of 2D structure of Ramelteon (green), 2-phenylmelatonin (orange) and Melatonin (yellow). (**B**) Structures of the MT_1_ complexed with RMT (green stick) and MT_2_ complexed with 2-PMT (orange stick) to 7/20 illustrate the binding site of the three binders.

To aid in examining and analyzing binder interactions, we schematically divided the binders into regions, as illustrated in Fig. 5. The carbon atoms are labeled from 1 to 19, and the rings are denoted by letters (A, B, and C). According to the Marvin Sketch analysis, neutral states were predominant (100%) at both pH values examined (7.0 and 7.4). In Fig. 5B, the MT_1_ and MT_2_ receptors are represented with the RMT and 2-PMT molecules, respectively, highlighting the binding site of the ligands.

Energy calculations and evaluations were performed for each of the six complexes, and convergence criteria were determined based on the variation of total energy (in kcal/mol) and radius r (in Å). The cumulative binding energy was obtained by summing up all the calculated energies between the residues within the radius and the binder. The analyses were continued until the contributions of amino acids from each radius were no longer significantly different from the total interaction energy found after each successive radius, with a threshold of less than 10%.

**Figure 6:**
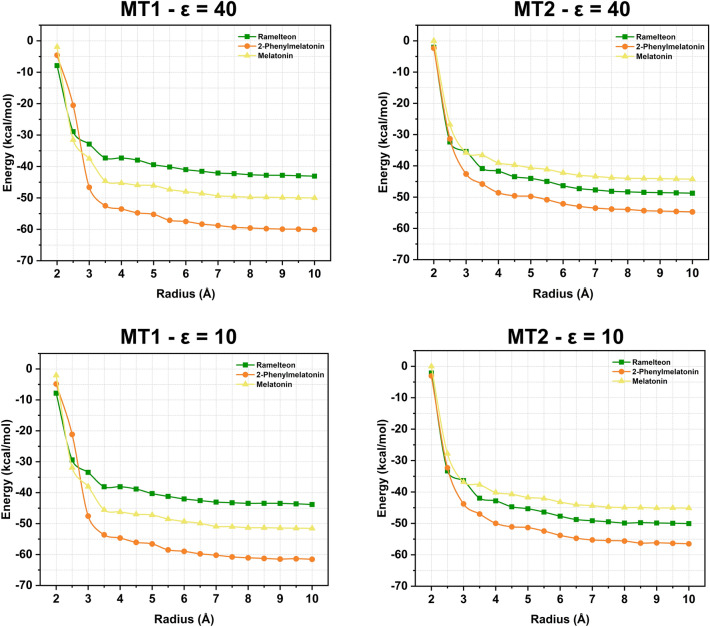
Plot representation of total interaction energy of MT_1_ and MT_2_ complexes for both dieletric constants (*ε* = 10 and *ε* = 40) as a function of the binding pocket radius calculated using the DFT/MFCC approach.

Figure 6 illustrates the change in total interaction energy with increasing radius. The convergence criteria are met between r = 7 Å and 8 Å for all complexes and dielectric constants (*ε* = 10 and *ε* = 40). However, calculations were conducted within r = 10 Å to ensure the comprehensive evaluation of crucial residues. As a result, for the MT_1_-RMT and MT_2_-RMT complexes, 96 and 105 residues were found to interact within r = 10 Å, respectively. In terms of affinity, the total interaction energy when *ε* = 40 (*ε* = 10) for MT_1_-RMT was − 43.08 kcal/mol (− 43.84 kcal/mol) and for MT_2_-RMT − 48.75 kcal/mol (− 50.07 kcal/mol), suggesting that the RMT drug has a slightly higher affinity for MT_2_-RMT. In the case of MT_1_-2-PMT and MT_2_-2-PMT, binding affinity was assessed for 104 and 108 residues, respectively. The total energies for these residues were − 60.09 kcal/mol (− 61.54 kcal/mol) for MT_1_-2-PMT and − 54.71 kcal/mol (− 56.49 kcal/mol) for MT_2_-2-PMT, when *ε* = 40 (*ε* = 10). For the MT_1_-MLT and MT_2_-MLT complexes revealed interactions with 86 residues within r = 10 Å. In terms of affinity, the total interaction energy for MT_1_-MLT was − 50.01 kcal/mol (− 51.53 kcal/mol) and for MT_2_-MLT was − 44.26 kcal/mol (− 45.13 kcal/mol), with dielectric constants *ε* = 40 (*ε* = 10).

Based on the data above, it is observed that the increasing order of affinity of the MT_1_ (MT_2_) receptor is: RMT < MLT < 2-PMT (MLT < RMT < 2-PMT), which suggests that 2-PMT acts better on both receptors compared to RMT and MLT. Moreover, all energy values show what is expected for *ε* = 10 compared to *ε* = 40, where the first shows lower values due to increased medium permittivity. Furthermore, it is observed that the pattern for *ε* = 10 and *ε* = 40 remained consistent in the plots, which strongly suggests that the DFT calculations were carried out correctly. Therefore, from now on, we will present the study with energy results focused on the dielectric constant *ε* = 40.

#### MT_1_ interaction analysis

**Figure 7:**
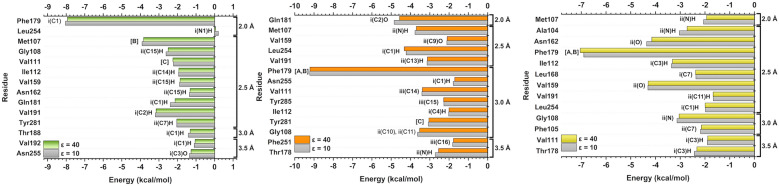
Graphic panels showing the most important residues for MT_1_ interaction with RMT (green bar), 2-PMT (orange bar) and MLT (yellow bar). Also, the region (i, ii or iii) and atom (based on Fig. 5 schematic representation) that interact with each residue at the binding site.

The individual energetic contribution of each amino acid was evaluated to understand the most significant residues in the interaction with MT_1_, as well as the ligand regions where these interactions occurred. In Fig. 7, the calculated energy of the key amino acids that contributed (both positively and negatively) to the interaction energy between the complexes can be observed. For interactions within the MT_1_-RMT complex (Fig. 7, green bar), the ten most important residues in decreasing order of affinity (in kcal/mol) are the following: Phe179 (− 7.93) > Met107 (− 3.83) > Val191 (− 3.15) > Gly108 (− 2.51) > Val111 (− 2.25) > Gln181 (− 2.13) > Tyr281 (− 2.04) > Ile112 (− 1.93) > Val159 (− 1.86) > Asn162 (− 1.32). Despite its very small energy value, Leu254 was the only residue shown in Fig. 7 to exhibit a negligible repulsive affinity (0.05 kcal/mol). For MT_1_-2-PMT (Fig. 7, orange bar), following the same order, these residues were: Phe179 (− 9.21) > Gln181 (− 4.57) > Leu254 (− 4.32) > Met107 (− 3.70) > Gly108 (− 3.53) > Val111 (− 3.39) > Val191 (− 3.13) > Tyr281 (− 3.05) > Thr178 (− 2.55) > Tyr285 (− 2.28) > Val159 (− 2.10) > Ile112 (− 2.01) > Phe251 (− 1.77) > Asn255 (− 1.71). In the case of MT_1_-MLT (Fig. 7, yellow bar), the main residues in descending order of affinity (in kcal/mol, for *ε* = 40) were: Phe179 (− 7.04) > Val159 (− 4.30) > Asn162 (− 4.15) > Ile112 (− 3.31) > Gly108 (− 3.05) > Ala104 (− 2.72) > Leu168 (− 2.38) > Thr178 (− 2.33) > Phe105 (− 2.14) > Leu254 (− 2.0) > Met107 (− 1.93) > Val111 (− 1.89) > Val191 (− 1.67). Seven common amino acid residues were observed between them: Phe179, Met107, Gly108, Val111, Val191, Val159, and Ile112. These residues are then suggested as key interacting residues, and main interactions with them will be evaluated in detail.

**Figure 8:**
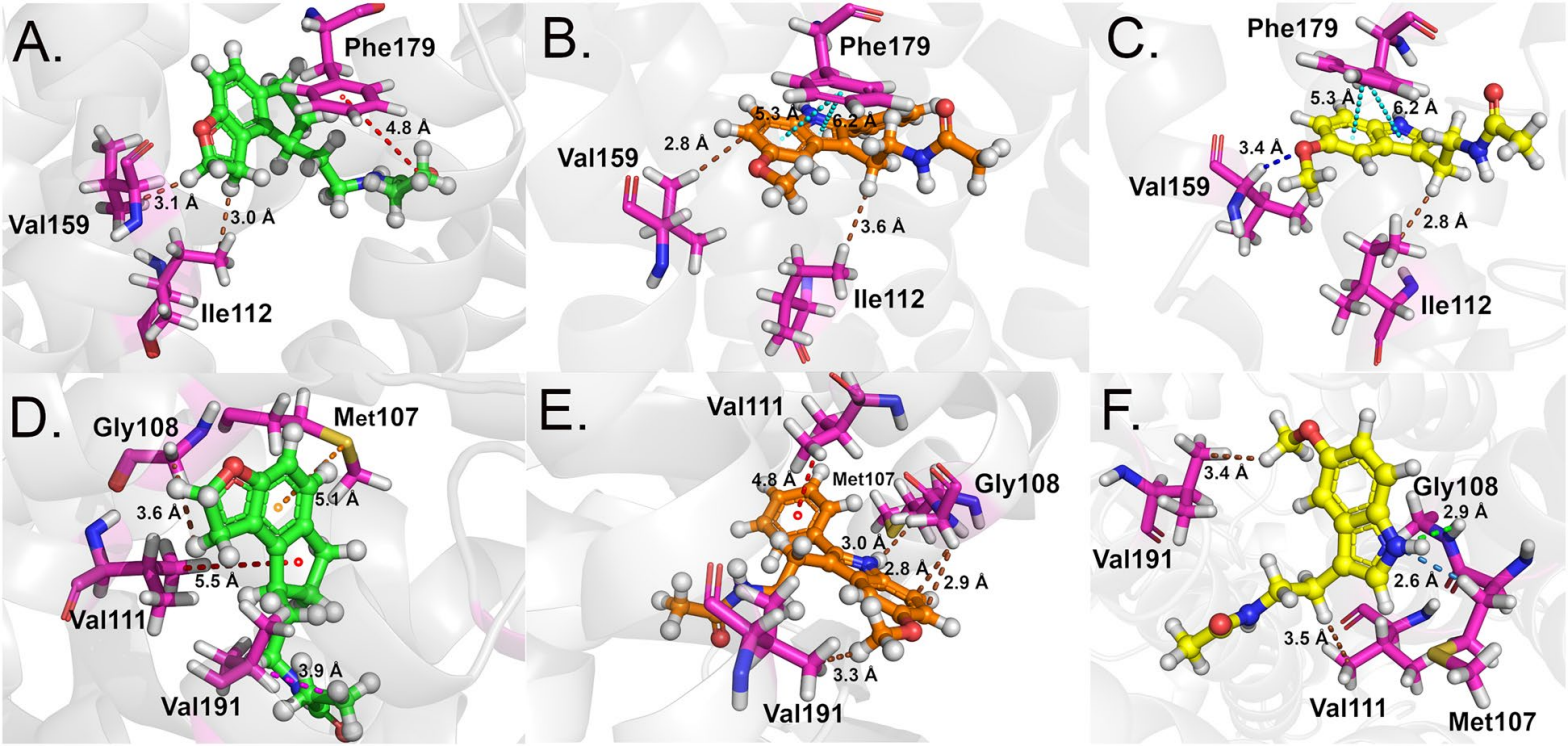
Tri-dimensional binding mode of RMT (**A** and **D**), 2-PMT (**B** and **E**) and MLT (**C** and **F**) in MT_1_ structure. The ligands are represented in ball and stick (RMT—green, 2-PMT—orange and MLT—yellow and the main MT_1_ amino acids in magenta sticks. The interaction types are colored as follows—green: hydrogen bond; cyan: pi–pi interaction; dark blue: non-conventional hydrogen bond; brown: dipole–dipole; red: pi-alkyl; orange: pi-sulfur.

Phe179 was the most important residue for interaction in all three MT_1_ complexes. It formed a pi-alkyl interaction with atom [ii(C8)] of the RMT molecule (Fig. 8A) and two pi-pi interactions with rings [A,B] of 2-PMT (Fig. 8B) and MLT (Fig. 8C). Interestingly, Phe179 showed high affinity for ligands (between − 7.04 kcal to − 9.21 kcal/mol) regardless of whether they interacted with its aromatic ring or aliphatic carbon. Val159 interacted with atoms from the aromatic region of RMT and 2-PMT (Fig. 8A-B) through dipole–dipole interactions. In contrast, it formed a non-conventional hydrogen bond with MLT (Fig. 8C), which resulted in a higher affinity interaction (− 4.30 kcal/mol) than the other molecules. Ile112 exhibited dipole–dipole interactions with all molecules. However, the interaction energies were lower for RMT (Fig. 8A) and 2-PMT (Fig. 8B) (− 1.93 kcal/mol and − 2.01 kcal/mol, respectively) than for MLT (Fig. 8C) (− 3.31 kcal/mol). This could be attributed to the distance factor, as Ile112 interacted with the same shared region of MLT (2.8 Å) and 2-PMT (3.6 Å). For RMT, the interaction involved a hydrogen atom bonded to a carbon atom of the aromatic ring [ii(C14)H], which may have affected the charge distribution and attractive forces.

Met107 was another important key residue for interaction with all ligands. It showed lower energy values for RMT (− 3.83 kcal/mol) (Fig. 8D) and 2-PMT (− 3.70 kcal/mol) (Fig. 8E) than for MLT (− 1.93 kcal/mol) (Fig. 8F). It formed a pi-sulfur interaction with ring [B] of RMT (Fig. 8D), a dipole–dipole interaction and a non-conventional hydrogen bond with atom [ii(N)H] of 2-PMT (Fig. 8E), and a dipole–dipole interaction with atom [ii(N)H] of MLT (Fig. 8F). RMT did not meet the non-conventional hydrogen bond parameters. Val111 performed pi-alkyl interactions with aromatic rings of RMT (Fig. 8D) and 2-PMT (Fig. 8E), which resulted in higher affinity than its dipole–dipole interaction with MLT (Fig. 8F). Gly108 exhibited considerable affinity for all ligands. It formed dipole–dipole interactions with atoms [ii(C15)H] of RMT (Fig. 8D), [ii(C10) and ii(C11)] of 2-PMT (Fig. 8E), and [ii(N)] of MLT (Fig. 8F). The latter was also a hydrogen bond. Finally, Val191 showed higher affinity for RMT (− 3.15 kcal/mol) and 2-PMT (− 3.13 kcal/mol) than for MLT (− 1.67 kcal/mol). It formed an alkyl-alkyl interaction with an atom [i(C2)H] of RMT and dipole–dipole interactions with atoms [ii(C13)H] of 2-PMT and [ii(C11)H] of MLT.

#### MT_2_ interaction analysis

**Figure 9:**
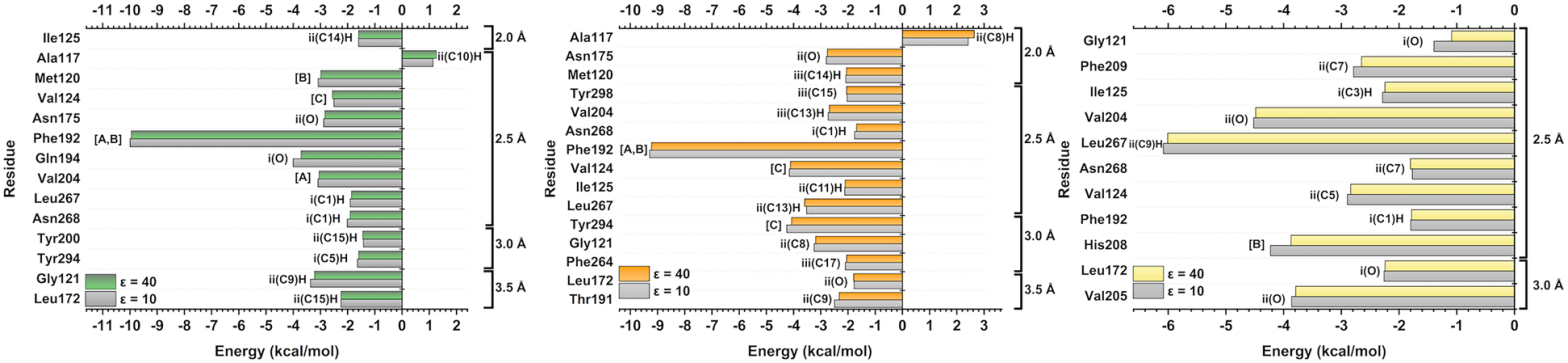
Graphic panels showing the most important residues for MT_2_ interaction with RMT (green bar), 2-PMT (orange bar) and MLT (yellow bar). Also, the region (i, ii or iii) and atom (based on Fig. 5 schematic representation) that interact with each residue at the binding site.

Among the residues of MT_2_-RMT complex (Fig. 9, green bar), the ten residues that most contributed to the binding affinity in decreasing order (in kcal/mol) were: Phe192 (− 9.95) > Gln194 (− 3.72) > Gly121 (− 3.22) > Val204 (− 3.05) > Met120 (− 2.99) > Asn175 (− 2.84) > Val124 (− 2.56) > Leu172 (− 2.25) > Asn268 (− 1.91) > Leu267 (− 1.86). Similar to MT_1_-RMT complex, only one residue (Ala117) showed repulsive energy (1.26 kcal/mol) for the binder. In the case of MT_2_-2-PMT (Fig. 9, orange bar), these residues are: Phe192 (− 9.22) > Val124 (− 4.12) > Tyr294 (− 4.07) > Leu267 (− 3.59) > Gly121 (− 3.19) > Asn175 (− 2.76) > Val204 (− 2.69) > Thr191 (− 2.32) > Ile125 (− 2.11) > Met120 (− 2.06) > Tyr298 (− 2.05) = Phe264 (− 2.05) > Leu172 (− 1.79) > Asn268 (− 1.69) > Ala117 (2.64). It is noteworthy that only the Ala117 residue in the MT_2_-2-PMT complex showed positive energy, indicating a repulsive interaction. When observing key residues in the MT_2_-MLT interaction (Fig. 9, yellow bar), the following amino acids were found to be significant for interaction energy (in kcal/mol, for *ε* = 40): Leu267 (− 6.01) > Val204 (− 4.48) > His208 (− 3.87) > Val205 (− 3.79) > Val124 (− 2.84) > Phe209 (− 2.65) > Ile125 (− 2.24) > Leu172 (− 2.24) > Asn268 (− 1.80) > Phe192 (− 1.79) > Gly121 (− 1.09). In common, six key residues were observed: Phe192, Val124, Gly121, Val204, Leu172, and Asn268.

**Figure 10:**
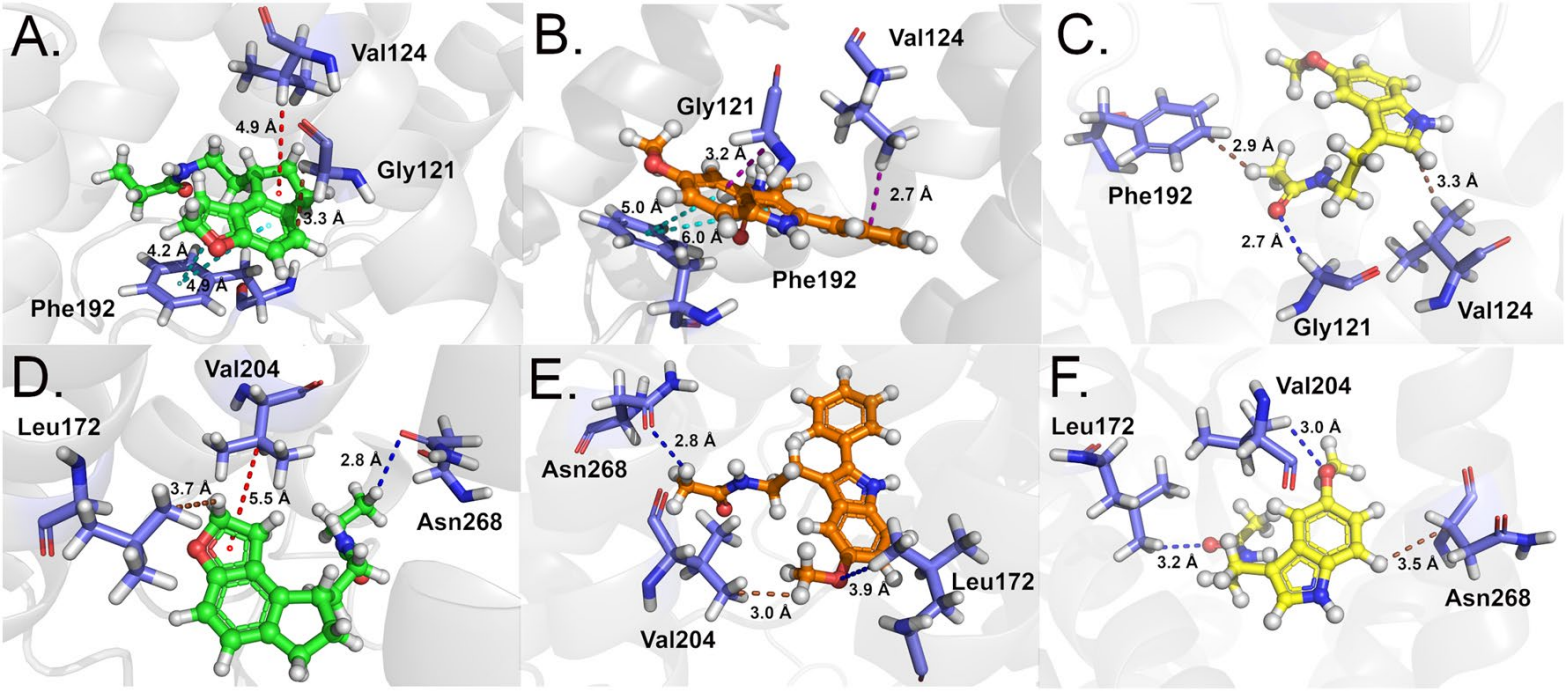
Tri-dimensional binding mode of RMT (**A** and **D**), 2-PMT (**B** and **E**) and MLT (**C** and **F**) in MT_2_ structure. The ligands are represented in ball and stick (RMT—green, 2-PMT—orange and MLT—yellow and the main MT_2_ amino acids in blue sticks. The interaction types are colored as follows—cyan: pi–pi interaction; dark blue: non-conventional hydrogen bond; brown: dipole–dipole; red: pi-alkyl; purple: pi-sigma.

As observed for MT_1_, Phe192 of MT_2_ structure had the highest affinity for RMT (− 9.95 kcal/mol) and 2-PMT (− 9.22 kcal/mol). It formed two pi-pi interactions with two aromatic rings of each ligand (Fig. 10A–B). In the MT_2_-MLT complex, Phe192 had a lower energy value (− 1.79 kcal/mol) and a dipole–dipole interaction with atom [i(C1)H] (Fig. 10C). Val124 performed a pi-alkyl interaction with RMT (− 2.56 kcal/mol), a pi-sigma interaction with the aromatic ring of 2-PMT (− 4.12 kcal/mol), and a dipole–dipole interaction with atom [ii(C5)] of MLT (− 2.84 kcal/mol) (Fig. 10A–C). Gly121 showed affinity energies lower than − 3.0 kcal/mol for RMT and 2-PMT (− 3.22 kcal/mol and − 3.19 kcal/mol, respectively). It formed a dipole- dipole interaction with atom [ii(C9)H] of RMT and a non-conventional hydrogen bond with atom [ii(N)] of 2-PMT (Fig. 10A,B). For MLT (− 1.09 kcal/mol), it formed a non-conventional hydrogen bond with atom [i(O)] (Fig. 10C).

Val204 had a higher affinity for MLT (− 4.48 kca/mol) than for RMT (− 2.69 kcal/mol) and 2-PMT (− 2.69 kcal/mol). It formed a pi-alkyl interaction with ring [C] of RMT (Fig. 10D), a dipole–dipole interaction with atom [iii(C13)H] of 2-PMT (Fig. 10E), and a non-conventional hydrogen bond with atom [ii(O)] of MLT (Fig. 10F). The latter favored its increased affinity. Asn268 formed non-conventional hydrogen bonds with atom [i(C1)H] of both RMT (− 1.91 kcal/mol) and 2-PMT (− 1.69 kcal/mol) (Fig. 10D,E). With MLT, it formed a dipole–dipole interaction with atom [ii(C7)], and the calculated energy was − 1.80 kcal/mol (Fig. 10F). Finally, Leu172 formed a dipole–dipole interaction with atom [ii(C15)H] of RMT (− 2.25 kcal/mol) (Fig. 10D) and non-conventional hydrogen bonds with 2-PMT (− 1.79 kcal/mol) and MLT (− 2.24 kcal/mol) (Fig. 10E,F).

### Alanine scanning

The alanine scanning study is an exciting approach to assessing the significance of amino acids in protein–ligand complexes. The PremPLI server, a straightforward tool based on machine learning, predicts the mutation’s effect solely using 3D structural information from the complex. In this case, the evaluated interactions involved the amino acids Gly108/Gly121, Val111/Val124, and Val191/Val204 across all ligands (RMT, 2-PMT, and MLT). The analysis of amino acids Gln181/194, Phe179/192, and Asn162/175 was restricted to those ranked with low energy according to QM/DFT calculations.

As seen in Table 3, the mutation with the most significant impact on interaction reduction was F179A/F192A. The highest quantum calculations energy for Phe192 was associated with the MLT ligand, and according to PremPLI, it also exhibited the most negligible impact on the mutation. Notably, among the amino acids Gly108/Gly121, Val111/Val124, and Val191/Val204, the G108A/G121A mutation had the most substantial impact on interaction, according to PremPLI. However, it is observed that in all complexes, the mutations resulted in positive ∆∆G values, indicating reduced affinity.

**Table 3:** Results of alanine scanning performed by the PremPLI server for the key amino acids indicated by QM/DFT calculations.

Protein	Ligand	Mutation	∆∆G	QM/DFT (kcal/mol)	Protein	Ligand	Mutation	∆∆G	QM/DFT (kcal/mol)
MT_1_	RMT	F179A	1.17	− 7.93	MT_2_	RMT	F192A	1.21	− 9.95
		Q181A	1.04	− 2.39			Q194A	0.99	− 3.72
		N162A	0.82	− 1.36			N175A	1.25	− 2.84
		G108A	0.99	− 2.61			G121A	1.03	− 3.22
		V111A	0.7	− 2.21			V124A	0.64	− 2.56
		V191A	0.63	− 3.19			V204A	0.6	− 3.05
	2-PMT	F179A	1.28	− 9.21		2-PMT	F192A	1.36	− 9.22
		Q181A	0.73	− 4.57			N175A	1.02	− 2.76
		G108A	1.07	− 3.53			G121A	0.93	− 3.19
		V111A	0.43	− 3.39			V124A	0.47	− 4.12
		V191A	0.54	− 3.13			V204A	0.44	− 2.69
	MLT	F179A	1.4	− 6.9		MLT	F192A	1.03	− 1.79
		N162A	0.71	− 4.36			G121A	0.85	− 1.09
		G108A	0.97	− 3.13			V124A	0.57	− 2.64
		V111A	0.21	− 1.9			V204A	0.66	− 4.48
		V191A	0.23	− 1.66					

Despite PremPLI’s results aligning with QM calculations, further robust analyses are essential to confirm the importance of amino acids Gly108/Gly121, Val111/Val124, and Val191/Val204 in ligand recognition and interaction with MT_1_ and MT_2_ receptors.

## Discussion

The search for new therapeutic strategies for the treatment of insomnia has become extremely important, mainly due to the various adverse effects caused by the use of BZDs and n-BZDs drugs in the general population. Several agonists and analogs of MLT have been studied for years, such as RMT, 2-iodomelatonin, agomelatonin, and 2-PMT^23^. However, all these drugs were developed before the three-dimensional resolution of the MT_1_ and MT_2_receptors, using the ligand-based drug discovery (LBDD) strategy.

Thanks to the recent advances in the resolution of the structures of the MT_1_ and MT_2_ proteins, it is now possible to meticulously evaluate the interactions between the receptors and their ligands. Therefore, this study was developed to fill two gaps related to the melatonin receptors: to obtain a model with MLT and its receptors and to evaluate the biochemical interactions with RMT, 2-PMT, and MLT to propose a structure-based drug discovery (SBDD) strategy, which is reported to be more complex and potentially better than LBDD^70^. The docking analysis allowed the orientation of MLT in the MT_1_ and MT_2_ receptors to be predicted in silico, since the structures of the experimental complexes are not yet available. Thus, it was possible to determine how MLT interacts with its receptors. The MD simulation provided important information regarding the stability of MLT in solution when bound to the MT_1_ and MT_2_ receptors. This is crucial because a molecule that does not stabilize in the active site of the protein with the conformational changes observed in solution does not become a suitable therapeutic target for use.

In this study, we can observe that MLT has good stability when complexed with its targets. In the docking and MD simulation study, it was possible to obtain complexes of MT_1_-MLT and MT_2_-MLT in their inactive state, as well as the other structures analyzed in this study. This comparison was feasible because the TM6 domain in the active form exhibits a displacement relative to the inactive form, which is well described by previous studies with experimentally resolved structures^69^.

In the QM/DFT analysis using the MFCC method, it was observed that the increasing order of affinity for MT_1_ was RMT < MLT < 2-PMT, which suggests that 2-PMT is the best ligand for MT_1_ activation and RMT is the weaker. In the case of MT_2_ this order was: MLT < RMT < 2-PMT, which suggests again 2-PMT as the best binder, however, in this case, MLT was ranked as the weaker MT_2_ ligand. All ligands showed higher affinity for MT_1_ compared to MT_2_, except the RMT binder. The 2-PMT and MLT results corroborate with in vitro experimental data where the affinity of 2-PMT and MLT for the receptors was evaluated. In these studies, it is possible to observe that the affinity of 2-PMT is higher than the affinity of MLT^71^ for both receptors, and both molecules have a higher affinity for MT_1_ than for MT_2_^71–73^. The RMT results do not agree with the experimental data. Further analysis should be performed to evaluate if this is a limitation of the calculation method or the experimental crystallographic data. However, most of the results shown here are in agreement with in vitro tests.

In the literature, the importance of three amino acids for protein interaction and activation is well described. These residues are Gln181/194, Phe179/192, and Asn162/175^23^. Undoubtedly, the most crucial key residue observed in this study was Phe179/192, which was found to be important in the interaction of all ligands with both proteins. The Gln181/Gln194 was found to be important for RMT (both proteins) and 2-PMT (MT_1_) interaction, and Asn162/Asn175 was found to be important for RMT (both proteins) and 2-PMT (MT_2_). Hence, the importance of Gln181/Gln194 and Asn162/175 depends on binder and protein, which is not true for Phe179/192. In addition to these amino acids, three other conserve amino acids—Gly108/Gly121, Val111/Val124, and Val191/Val204—were shown to play important roles in the interaction with MT_1_ and MT_2_ for all binders. Beyond Gln181/194, Phe179/192, and Asn162/175 amino acids, only Thr178 and Met107 were recently observed in other studies involving binding with this protein^73^. Thus, we have potential amino acids crucial for protein–ligand interactions with both receptors. This could lead to more optimized molecule development based on the SBBD approach since key residues for the interaction of these molecules were identified, as well as the regions of the melatonin analogs that favored an increased affinity for the receptors. This is an important, although initial, step towards solving the issue of the short half-life of melatonin receptor ligands.

In conclusion, recent advances in deciphering the structures of MT_1_ and MT_2_ proteins have allowed for a detailed exploration of interactions with their binders. Molecular docking and dynamics simulations were employed to generate MT_1_-MLT and MT_2_-MLT complexes, revealing stronger binding to MT_1_ than MT_2_. Notably, 2-PMT displayed higher affinity for both receptors compared to MLT, aligning with experimental findings. The analysis highlighted the importance of specific amino acids, such as Gln181/194, Phe179/192, and Asn162/175, in protein-ligand interactions, providing insights into potential therapeutic strategies. Also, it described new important interactions with Gly108/Gly121, Val111/Val124, and Val191/Val204.

This study enhances our understanding of receptor-ligand interactions and offers implications for future drug development. All the analyses of the interactions described here and the key regions of the receptors and ligands involved in the interactions are extremely relevant information for developing more effective drugs for treating sleep disorders. However, it is known that analyses involving drug optimization and mutation analysis are necessary to confirm the research data and fill these gaps. Future studies should consider the virtual screening of molecules with the important regions of the ligands (especially the aromatic rings) to find new ligands with greater affinity and optimize existing molecules by adding and/or removing groups that favor/disfavor interaction affinity.

## Methods

### Molecular docking and molecular dynamics simulation

Since there is no crystallized structure of the MT_1_ and MT_2_ proteins with MLT, the first step of the study involved obtaining the complexed structures through molecular docking using the AutoDock Vina program (referred to as Vina here)^68^. Initially, the three-dimensional (3D) structure of MLT was obtained from the PubChem website (www.pubchem.ncbi.nlm.nih. gov/compound/896) in SDF format. The molecule’s protonation at pH = 7.0 and 7.4 (following the protein experimental pH) was verified using the MarvinSketch code version 17.24 (Marvin Beans Suite – ChemAxon, www.chemaxon.com). Subsequently, the molecule was converted to PDBQT format using the OpenBabel^74^ server.

The MT_1_ structure was obtained from the Protein Data Bank (www.rcsb.org). Four MT_1_ crystals were compared for resolution, with codes 6ME2, 6ME3, 6ME4, and 6ME5. The crystal with the lowest resolution (6ME2^25^—2.8 Å), which suggests higher quality, was selected for MLT docking. The same procedure was followed for the MT_2_ structure, where structures 6ME6, 6ME7, 6ME8, and 6ME9 were compared. The 6ME6^23^ structure was chosen due to its lower resolution (2.8 Å) among MT_2_ structures.

Before converting to the PDBQT format, the MT_1_ and MT_2_ proteins underwent a cleaning step, chain adjustments, and minimization on the Discovery Studios server. In this case, artifacts from the crystallization process were removed, as well as the ligands RMT and 2-PMT, bound to MT_1_ and MT_2_, respectively. Additionally, missing side chains and hydrogen atoms were added. The protonations of the MT_1_ and MT_2_ proteins were evaluated on the online PropKa server for pH 7.0 and 7.4, respectively, in accordance with the crystallization experiment. After these modifications, the protein backbone’s conformations were restrained, and an energy minimization (EM) step was performed using the CHARMm (Chemistry at Harvard Molecular Mechanics) force field. The EM utilized convergence tolerances of 10^*−*5^ kcal/mol for total energy change, 10^*−*3^ kcal/mol for the mean square root of the RMS gradient, and 10^*−*5^ Å for the maximum atomic displacement, employing the Smart Minimizer algorithm. For the conversion of large molecules from PDB to PDBQT format, AutoDock Tools^75^ was used.

The same binding sites of RMT and 2-PMT were considered for melatonin anchoring. For each system, the melatonin molecule was docked 1000 times, each time generating 10 binding conformation modes. The best conformation (lower Vina score) of each system was selected to proceed to MD simulations.

MD simulations were conducted to optimize the binder conformation within the binding pocket. Independent triplicate simulations were performed using GROMACS 2022 software^81^. The ligand parameters for the MD simulations were obtained using the ACPYPE server (www.bio2byte.be/acpype/)^76^ with Gasteiger as the charge method and GAFF2 as the force field. The server provided all the necessary topology and parameter files for MD simulations using GROMACS software. The force field selected for protein was the Amberff99SB-ILDN. Six MD simulations were carried out for the protein-ligand complex, three for each MT_1_ and MT_2_ systems.

For each system, a cubic box was utilized with the TIP3P water model extended 12 Å away from solute atoms, and Cl^-^ ions were added to neutralize it. Two rounds of energy minimization were executed to adjust unfavorable contacts in the initial structure. The first minimization step involves a maximum of 20000 steps or until the maximum force on any atom is reduced to below 50 kJ/mol/nm. The steepest descent algorithm was employed with protein restraint to focus on solvent relaxation. The second minimization step, without protein restraint, was performed in flexible water using the same steepest descent algorithm, and the maximum steps were increased to 10,000 or until the force on any atom fell below 250 kJ/mol/nm.

The system’s pressure and temperature were adjusted to 1 atm and 310 K, in two separate 100 ps steps, referred to as the NVT ensemble (temperature setting) and NPT ensemble (pressure setting). The modified Berendsen^77^ and Parrinello-Rahman^78^ algorithms were applied to control the system temperature and pressure, respectively. Throughout both steps, hydrogen bonds were constrained using the LINCS algorithm^79^, and positional restraints were applied to the protein to stabilize the solvent around the solute.

Long-range electrostatic interactions were computed using the Particle Mesh Ewald (PME) summation method, employing a non-bonded interaction cut-off of 1 nm. The equations of motion were integrated using the leap-frog algorithm^80^ with a time step of 0.2 fs. Before the MD simulation, a small NPT ensemble of 1 ns was conducted without any restrictions on protein position, followed by a production run for 200 ns without restrictions on protein conformation. The MD run produced a total of 2000 protein frames.

The gmx_MMPBSA program^81^ was employed to analyze the last 500 frames (50 ns) of the complexes for each MD simulation using hybrid Quantum Mechanics/Molecular Mechanics—Generalized-Born surface area (QM/MM-GBSA) free energy calculation. The QM region was limited to residues near 5 Å from the binder, and the applied semi-empirical functional was PM6-DH + . Solvent molecules and Cl^-^ ions were excluded from the analysis. This method allowed the calculation of interaction energy for various conformations of the biological complex at a relatively low computational cost, enabling the selection of complexes with higher affinities for a more robust and accurate analysis using QM/DFT. Hence, the complexes with lower energy for each system were chosen for further QM (DFT) calculations using the MFCC approach.

The MD trajectories were visualized using UCSF Chimera software^82^. Root mean square deviation (RMSD) and fluctuation (RMSF) were calculated using the "gmx" commands of the GROMACS package. All plots were generated using the R language in RStudio 4.1.1 (http://www.rstudio.com/), and protein image representations were created using PyMol^83^.

### MFCC and quantum mechanical calculations

The QM calculation is an accurate methodology for studying ligand–protein interactions. However, it has been observed that the computational cost is significantly high for large systems. As the present study aims to analyze protein–ligand interactions, and therefore, is considered a complex biological system for QM calculations, the MFCC methodology developed by Zhang and Zhang^71^ was applied to each of these systems to overcome this limitation. The MFCC scheme involves splitting the protein into individual amino acids by breaking the peptide bonds and calculating the interactions between each residue and the ligand separately. The summation of individual amino acid energies provides an approximate binding energy.

The MFCC approach employs "caps" to complete the valence of the amino acids after breaking the peptide bonds at both the N- and C-termini. These caps are composed of amino acid residues that precede and succeed the main amino acid. Additionally, the caps serve the purpose of closely reproducing the amino acid environment. Consequently, it becomes possible to calculate an approximate binding affinity for large systems, such as a protein-ligand complex.

Equation (1) presents the MFCC scheme for calculating the interaction energy (IE(BID/R^i^)) between the binder (BID) and the amino acid R^i^, with i denoting the i^th^ amino acid of the protein chain.:

1 $$IE\left( {BID/R^{i} } \right) = E(BID + C^{i - 1} R^{i} C^{i + 1} ) - E\left( {C^{i - 1} R^{i} C^{i + 1} } \right) - E(BID + C^{i - 1} C^{i + 1} ) + E\left( {C^{i - 1} C^{i + 1} } \right)$$

The caps, represented as C^i−1^ and C^i+1^, correspond to the neighboring residues covalently bonded to the amine and carboxyl groups of R^i^, respectively. The first term (*E*(*BID* +*C*^*i−*1^*R*^*i*^*C*^*i*+1^)) calculates the interaction energy between the binder BID and the main residue (R^i^) bonded to the caps (C^i-1^ and C^i+1^).

However, MFCC aims to assess the individual amino acid contributions to the binding affinity. Thus, the second and third terms are introduced to isolate the contribution of the main residue and eliminate the interaction energy between the binder and the caps. The second term (*E*(*C*^*i−*1^*R*^*i*^*C*^*i*+1^)) represents the energy of the residue (R^i^) bonded to the caps (C^i-1^ and C^i+1^), while the third term (*E*(*BID* +*C*^*i−*1^*C*^*i*+1^)) indicates the interaction energy between the BID and the caps (C^i-1^ and C^i+1^). Subtracting the energies calculated in the second and third terms from the first term removes their influence on the energy interaction between BID and R^i^.

Since the caps’ energies were subtracted twice (in the second and third terms), it becomes necessary to add them back in the fourth term (*E*(*C*^*i−*1^*C*^*i*+1^)) to account for their effect on the energy interaction between BID and R^i^. This step accurately evaluates the individual amino acid’s contribution to the overall binding affinity.

For the MFCC involving the MLT molecule, the frames with the lowest energy obtained from the QM/MM-GBSA analysis of MT_1_ and MT_2_ were selected. No modification was needed since the structures were adjusted previously. As for the 2-PMT molecule, the structures were obtained from the PDB, where the structures of MT_1_ and MT_2_ were submitted under accession codes 6ME3^25^ and 6ME6^23^, respectively. The crystallographic structures of the MT_1_-RMT and MT_2_-RMT complexes were taken from the PDB (PDB codes: 6ME2^25^ and 6ME9^23^, respectively). As mentioned earlier, structural adjustments are necessary to address potential limitations of the X-ray technique. Thus, the same cleaning procedure, protonation verification at pH 7.0 (MT_1_) and 7.4 (MT_2_) (for both ligands and receptors), the addition of missing side chains, and energy minimization, as performed previously, were applied to these structures. The only difference is that the 2-PMT and MLT molecules were retained in the structures.

After performing fragmentation for each amino acid, the interaction energy between the receptor and the binder was computed using the Gaussian 16 package^84^, which employs the density functional theory (DFT) formalism^85,86^. The simulations were carried out using the generalized gradient approximation (GGA) with the B97D functional, which has been proven to be an efficient and accurate QM method for large systems, particularly when dispersion forces play a significant role^87^. To represent the Kohn-Sham orbitals for all electrons, a small basis set 6-311+G(d,p) with triple split valence (valence triple-zeta), an additional diffuse function (+), and polarization functions (d,p) were applied. The conductor-like polarizable continuum model (CPCM) was utilized to account for solvent effects in the QM calculations, using dielectric constants (*ε*) of 10 and 40. The constant of 40 is reported as being similar to the crystalline environment^57,58,88^. Meanwhile, the constant of 10 is used here as a control, where a lower constant is expected to result in a higher medium permissivity and lower energy values. Thus, we can ensure that calculations for both constants were performed correctly. Each term of Equation (1) was obtained from the DFT simulations.

It has been established that as the amino acids are increasingly distant from the binder, their contribution to the binding energy with the small molecule is reduced. To eliminate the calculation of unimportant interactions, a convergence analysis of the total binding energy was conducted to limit the number of amino acid residues considered. The analysis incorporated the closest residues from the binding site and excluded the most distant ones. The interaction energy of amino acid residues within imaginary spheres with a pocket radius of r centered at the binder was evaluated, where r = R/2 (for R = 1, 2, 3, 4,..., N_n_; with N_n_ being the next natural number of sequences)^88^. The convergence criteria were met when the total energy, while increasing the radius r, did not change by more than 10% compared to the previous radius value.

### Alanine scanning

To verify the significance of amino acids in protein–ligand interactions, the PremPLI server was utilized to conduct an alanine scanning study, wherein the original amino acid was substituted with alanine. This server employs machine learning through a random forest algorithm, with training data based on experimental data from 796 mutations across 360 protein–ligand complexes^89^.

Consequently, the server can predict the effect on protein–ligand interaction using ∆∆G, where positive values correspond to decreased interaction and negative values indicate increased interaction.

In the present study, the analysis focused on the amino acids Gly108/Gly121, Val111/Val124, and Val191/Val204 for all ligands. The analysis of amino acids Gln181/194, Phe179/192, and Asn162/175 was limited to those ligands that exhibited strong interaction.

### Supplementary Information

Supplementary Figures.

## Data Availability

The datasets analyzed during the current study are available in the Protein Data Bank repository, following the web links: https://www.rcsb.org/structure/6ME2, https://www.rcsb.org/structure/6ME3, https://www.rcsb.org/structure/6ME6, https://www.rcsb.org/structure/6ME9, https://www.rcsb.org/structure/7VH0 and https://www.rcsb.org/structure/7DB6. Additional datasets used and/or analyzed during the current study are available from the corresponding author upon reasonable request.
