# Supplementary material for: Quantum mechanics insights into melatonin and analogs binding to melatonin MT1 and MT2 receptors
Source: Sci Rep. 2024 May 13;14:10922. doi: 10.1038/s41598-024-59786-x (PMC11091226; doi:10.1038/s41598-024-59786-x)
Supplement: Supplementary file 1 — Supplementary Figures. [file 41598_2024_59786_MOESM1_ESM.docx]

**Title:** Quantum Mechanics Insights into Melatonin and Analogs Binding to Melatonin MT_1_ and MT_2_ Receptors

**Journal:** Scientific Reports

**Authors:** Gabriela de Lima Menezes, Katyanna Sales Bezerra, Jonas Ivan Nobre Oliveira, John Fontenele Araújo, Douglas Soares Galvão, Roosevelt Alves da Silva, Marielena Vogel Saivish, and Umberto Laino Fulco.

**Corresponding Author:** Umberto Laino Fulco

Supplementary Material


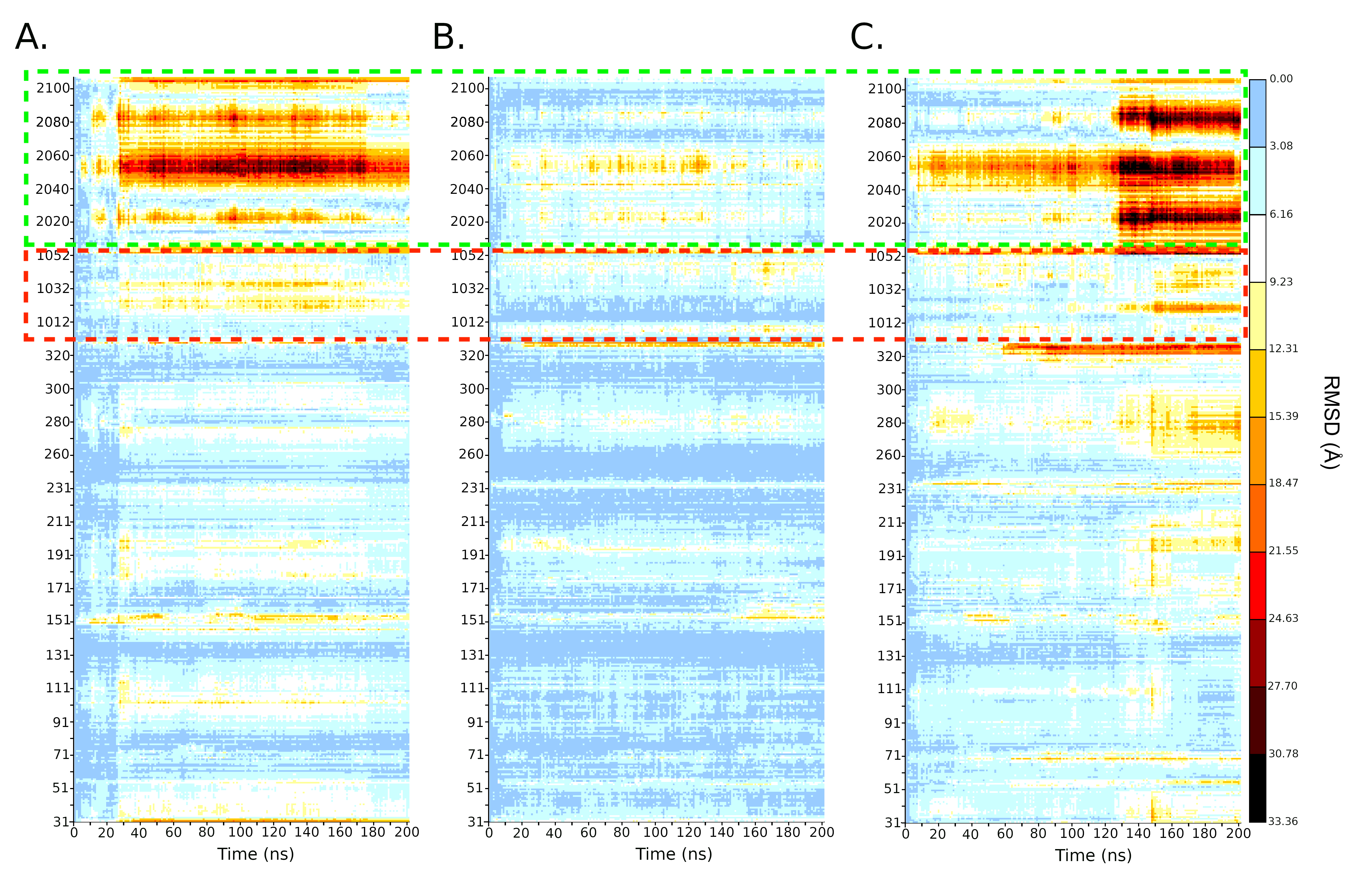


Supplementary Figure S1 – Heatmap of root mean square deviation (RMSD) for each residue over time in three replicates of the molecular dynamics simulation of MT_2_-MLT complex. The MT_2_ receptor consists of residues 31 to 327. The fusion protein is composed of rubredoxin (residues 1000 to 1052, red dashed rectangle) and apocytochrome BRIL (residues 2001 to 2106, green dashed rectangle). The figure shows that the fusion protein regions have higher fluctuations than the MT_2_ receptor. A three-dimensional representation of the complex is shown in Supplementary Figure S2.


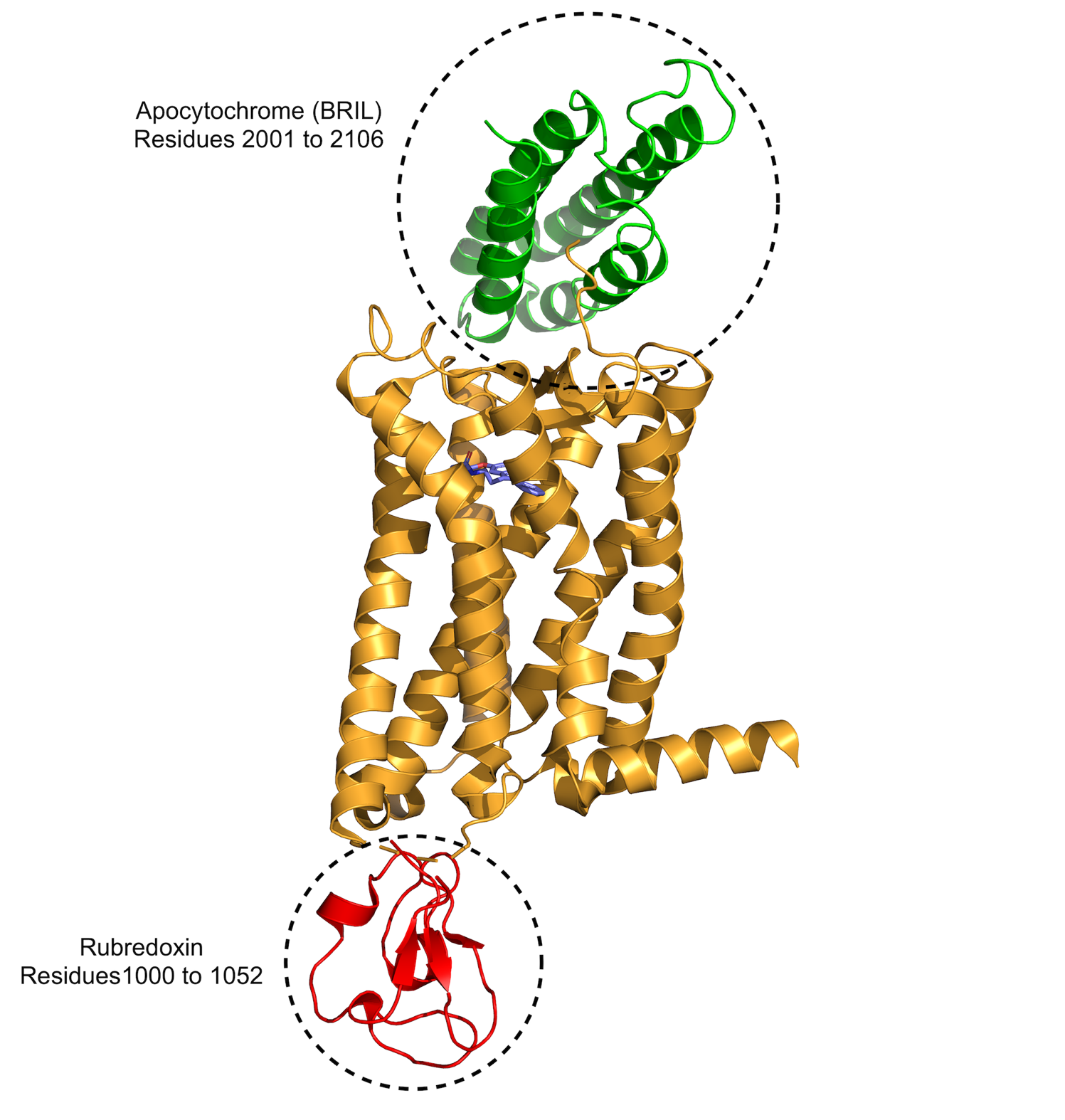


Supplementary Figure S2 – Three-dimensional structure of MT2 receptor in complex with fusion proteins. The structure was obtained from the Protein Data Bank (PDB ID: 6ME6). The rubredoxin protein is shown in red cartoon, the apocytochrome BRIL protein is shown in green cartoon, and the MT2 receptor is shown in orange cartoon. The 2-phenylmelatonin ligand is shown in blue stick.


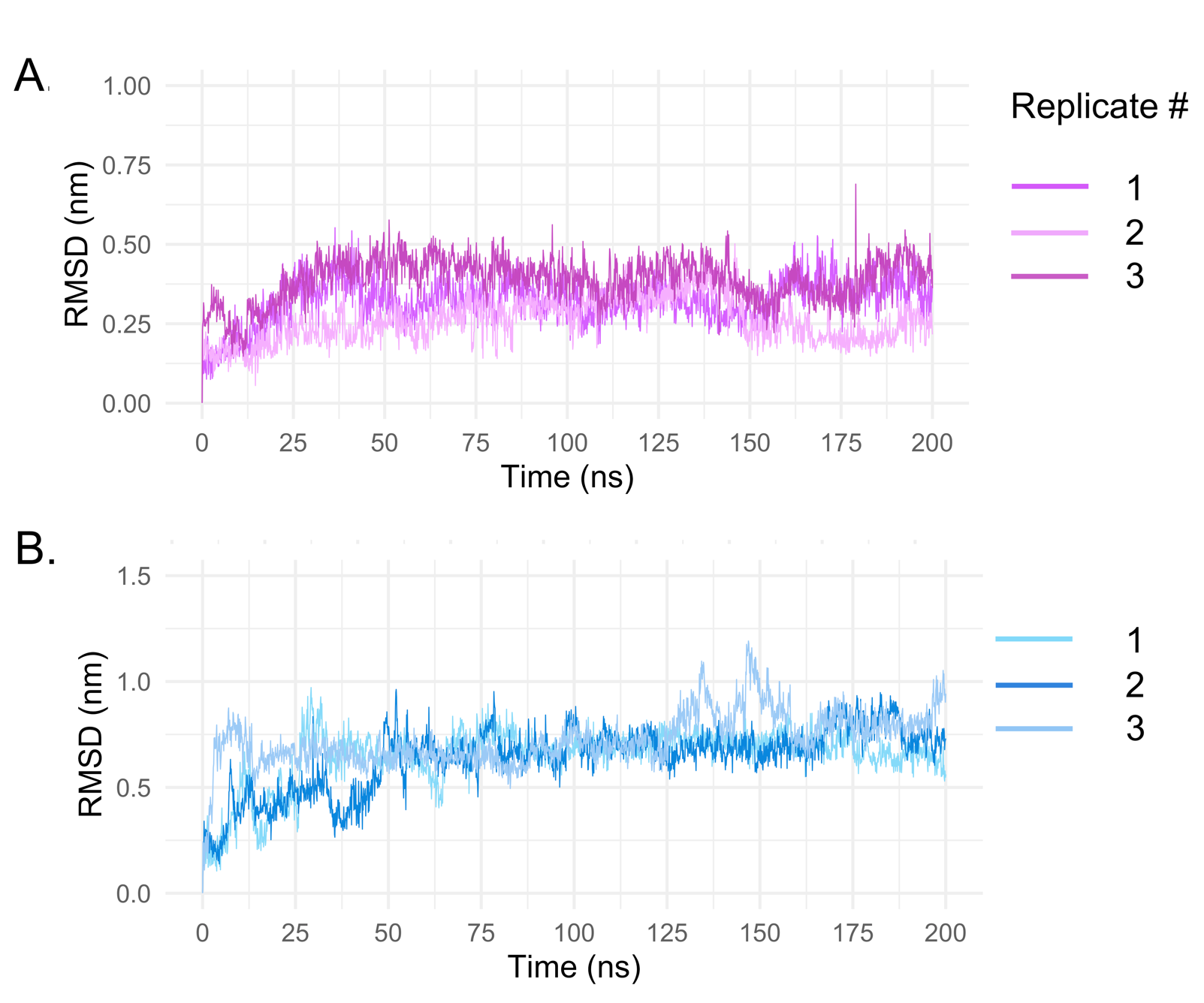


Supplementary Figure S3 – Root mean square deviation (RMSD) of melatonin ligand over time in MT1 (A) and MT2 (B) complexes. The figure shows the RMSD values for each of the three replicates of the molecular dynamics simulations for both complexes.
